# Association between Albumin-to-Globulin Ratio and Periodontitis: A Cross-Sectional and Mendelian Randomisation Analysis

**DOI:** 10.3290/j.ohpd.c_2765

**Published:** 2026-07-27

**Authors:** Jiakui Wang, Pengxian Xie, Zhengqiang Kang

**Affiliations:** a Jiakui Wang Dentist, Department of Stomatology, Cangzhou People’s Hospital, No. 13 Qingchi North Avenue, Cangzhou, Hebei Province, 061000, China. Researched, designed, conducted the study, wrote and reviewed the manuscript.*; b Pengxian Xie Master’s student, College of Stomatology, North China University of Science and Technology, Tangshan, Hebei Province, 063000, China. Researched, designed, conducted the study, wrote and reviewed the manuscript.*; c Zhengqiang Kang Dentist, Department of Stomatology, Cangzhou People’s Hospital, No. 13 Qingchi North Avenue, Cangzhou, Hebei Province, 061000, China. Researched, designed, conducted the study, wrote and reviewed the manuscript.

**Keywords:** albumin-to-globulin ratio, Mendelian randomisation analysis, NHANES, periodontitis

## Abstract

**Purpose:**

Periodontitis has been linked to systemic inflammation and nutrition. The albumin-to-globulin ratio (AGR) is a readily available inflammatory and nutritional index, but its relationship with periodontitis remains to be fully clarified. This study examined the association between AGR and periodontitis using the National Health and Nutrition Examination Survey (NHANES) data and Mendelian randomisation (MR).

**Methods and Materials:**

The cross-sectional analysis included 10,094 participants from NHANES 2009–2014. Weighted multivariable logistic regression, restricted cubic splines (RCS), subgroup analyses, and threshold effect analysis were performed. Furthermore, MR analysis was conducted using genetic data for AGR (98,626 individuals) and periodontitis (9,560 cases and 169,166 controls) to further evaluate the relationship.

**Results:**

After fully adjusting for confounders, logistic regression shows elevated AGR was associated with lower odds of periodontitis (OR = 0.52, 95% CI: 0.40–0.67). The top quartile group had 44% fewer cases of periodontitis than the lowest quartile group (OR = 0.56, 95% CI: 0.46–0.68). The RCS revealed an L-shaped nonlinear association between AGR and periodontitis. Threshold effect analysis showed that when AGR < 1.79, higher AGR was significantly associated with lower odds of periodontitis (OR = 0.37, 95% CI: 0.28–0.50). Stratified analyses suggested a stronger association among participants aged < 60 years. MR did not substantiate a genetic correlation between AGR and periodontitis (*p* = 0.304).

**Conclusion:**

Higher AGR was associated with a lower prevalence of periodontitis, particularly among adults aged < 60 years. However, MR analysis did not provide genetic evidence supporting this link. Prospective studies are needed to clarify this relationship.

Periodontitis represents an important component of the global burden of oral diseases, with severe periodontitis affecting more than one billion people worldwide and serving as one of the major causes of tooth loss in adults.^24^ The initiation and progression of periodontitis are mainly driven by a sustained interaction between plaque biofilm and host immune response, ultimately leading to clinical attachment loss, alveolar bone destruction, and tooth loss.^1^ Increasing evidence indicates that periodontal inflammation is not confined to periodontal tissues, but may also influence the systemic circulation through the dissemination of periodontal pathogens, the release of inflammatory mediators, and activation of the acute-phase response, thereby providing a biological basis for the associations between periodontitis and systemic diseases such as cardiovascular disease and chronic kidney disease.^14,33,34^ Therefore, in addition to conventional periodontal clinical measurements, blood–derived indicators reflecting host inflammatory status and nutritional status may help characterise the systemic features of periodontitis and provide additional evidence for periodontal risk assessment.

The serum albumin-to-globulin ratio (AGR) has drawn major interest in recent years as a thorough predictor of the individual’s nutritional state and degree of inflammation. Albumin, synthesised by the liver, is the primary protein found in plasma and serves critical physiological roles, including maintaining colloid osmotic pressure, substance transport, and antioxidant.^4^ In inflammatory states, serum albumin concentrations diminish due to inhibited hepatic production and heightened vascular permeability.^32^ Chung and Chan found that older age and lower serum albumin levels were associated with periodontitis.^11^ Globulins, which mainly consist of immunoglobulins and acute-phase reactive proteins, have significantly higher concentrations in infected and inflammatory states.^40^ Rad et al^28^ found that the concentration of globulins in the gingival sulcus fluid of chronic periodontitis sufferers was markedly elevated relative to normal controls. This finding suggests that globulin, a crucial component of the immune response, participates in the inflammatory process in periodontal tissues.

Compared with a single protein indicator, AGR integrates the trends of two types of proteins and can reflect the inflammatory-immune status of the body more comprehensively and sensitively.^10^ Zeng et al^42^ demonstrated that a decrease in periodontal probing depth (PD) was greatly related to a drop in total protein, which was accompanied by an increase in AGR. Moreover, several studies have confirmed that AGR may function as a prospective marker to evaluate disease severity and prognosis in cancer, diabetes, and inflammatory diseases.^35,37,39^

Despite the value of AGR in reflecting systemic inflammatory status, its relationship with periodontitis remains incompletely understood.^8,15^ Periodontitis is a chronic inflammatory disease in which the local inflammatory response not only poses risks to oral health but may also affect multiple organs and systems through systemic inflammatory burden.^16^ Previous studies have examined individual serum proteins, serum acute-phase biomarkers, and AGR in relation to periodontitis.^8,15,30^ Building on this work, further evaluation of AGR in periodontitis may provide complementary information for periodontal risk assessment and clinical management.

Therefore, we hypothesised that lower AGR levels would be associated with higher odds of periodontitis. The first aim of this study was to evaluate the observational association between AGR and periodontitis among United States (US) adults using data from the National Health and Nutrition Examination Survey (NHANES). The second aim was to further examine, through two-sample Mendelian randomisation (MR) analysis, whether genetically predicted AGR was associated with periodontitis.

## METHODS AND MATERIALS

### NHANES Study

#### Survey description

NHANES is designed to assess the health and nutritional status of the US civilian noninstitutionalised population. The survey is conducted every two years using a multistage stratified probability sampling procedure to obtain a nationally representative sample.^22^

This study plan has been approved by the Ethics Review Committee of the National Centre for Health Statistics. Every subject furnished signed documented consent before participation, confirming their full comprehension of the study objectives, processes, and potential hazards. The 2009–2010 cycle was approved as a continuation of Protocol #2005-06, the 2011–2012 cycle was approved under Protocol #2011-17, and the 2013–2014 cycle was approved as a continuation of Protocol #2011-17.^7^ As this study utilised only de-identified and publicly available NHANES data, additional ethical approval was not required. The writing of this study followed the requirements of the guidelines for cross-sectional studies in STROBE.^13^

#### Study population

This investigation utilises data from three cycles of the NHANES conducted between 2009 and 2014, as that period contains the most recent periodontal examination information. Initially, 30,468 participants were included. Participants were eligible for inclusion if they met the following criteria: (1) aged 30 years or older; (2) completion of a standardised periodontal examination; and (3) availability of data required for the calculation of AGR. The exclusion criteria were as follows: (1) pregnancy; (2) incomplete periodontal examination data; (3) missing AGR data; and (4) AGR outliers identified according to the 3-fold interquartile range (IQR) criterion. For missing data in covariates, the JOMO package was employed to conduct multiple imputation. After the above screening procedures, 10,094 subjects were finally incorporated into the analysis of this study. The detailed participant selection process is shown in Figure 1.

#### Calculation of AGR

Serum albumin concentration was determined using a DxC800 fully automated biochemical analyser. The instrument utilises a two-colour digital endpoint method for quantitative analysis by observing the variation in absorbance at 600 nm of the binding product of albumin to Bromocresol Purple. The formula for AGR is as follows:

AGR = serum albumin (g/L) / serum globulin (g/L).

For categorical analyses and trend tests, AGR values were ranked in ascending order and categorised into four approximately equal-sized groups using a quartile-based grouping method. The resulting AGR quartile ranges were as follows: Q1 (0.39, 1.32), Q2 (1.32, 1.50), Q3 (1.50, 1.69), and Q4 (1.69, 2.78).

#### Case definition of periodontitis

The case definition of periodontitis was based on the 2012 Centres for Disease Control and Prevention/American Academy of Periodontology (CDC/AAP) criteria.^12,26^ Periodontitis status was classified using two core clinical indicators, clinical attachment loss (CAL) and PD, together with the distribution of affected teeth and sites. The specific classification criteria were as follows:

Mild periodontitis: at least two neighbouring sites with CAL ≥ 3 mm and at least two neighbouring sites with PD ≥ 4 mm; or presence of a single site with PD ≥ 5 mm.
Moderate periodontitis: at least two neighbouring sites that are not on the same tooth have a CAL ≥ 4 mm, or at least two neighbouring sites have a PD ≥ 5 mm.
Severe periodontitis: CAL ≥ 6 mm on at least two non-identical teeth and PD ≥ 5 mm on at least one adjacent site.
No periodontitis: does not meet any of the above diagnostic criteria for periodontitis.

In the present study, participants were further divided into two groups: the non-periodontitis group and the periodontitis group. Participants diagnosed with mild, moderate, or severe periodontitis were classified into the periodontitis group, whereas those with no periodontitis were classified into the non-periodontitis group, in line with previous studies.^9^

#### Selection of covariates

This study considered several covariates, including sociodemographic factors such as age, gender, race, educational attainment, marital status, and the poverty-to-income ratio (PIR). Additionally, personal habits such as smoking, alcohol use, and physical activity were included, along with body mass index (BMI) and chronic conditions, including diabetes, hypertension, cardiovascular disease (CVD), chronic kidney disease (CKD), and rheumatoid arthritis (RA). Refer to Supplementary Table S1 for the classification and diagnostic criteria for these variables.

### MR Study

#### Survey description

The research employed a two-sample MR technique to investigate causal relationships between AGR and periodontitis, with the complete study flow illustrated in Figure 2. Single-nucleotide polymorphisms (SNPs) employed in MR analysis must satisfy three principal assumptions^5^ : (1) they must be related to exposure variables; (2) they should be unaffected by confounders influencing both exposure and outcome; and (3) SNPs must affect the outcome exclusively through the exposure, without alternative causal pathways. No further ethical approval or informed permission was necessary as the data utilised were publicly accessible. The investigation was conducted in strict adherence to the STROBE-MR standards to guarantee its scientific validity and rigour.^31^

#### Data sources

Because the research sample was all East Asian, bias resulting from ethnic differences was effectively avoided. Both the AGR and periodontitis data were taken from publicly available genome-wide association studies (GWAS) pooled data. The IEU OpenGWAS database (ID: bbj-a-4) provided the AGR data, which included 98,626 samples and 6,108,953 SNPs.^17^ Data on periodontitis, including 9,560 patients in the case group and 169,166 in the control group, were taken from the NHGRI GWAS Catalog database.^29^

#### Screening of SNPs

SNPs that were strongly linked to AGR (*p* < 5 × 10^–8^) were chosen, and their F-statistics were computed to make sure the F value was higher than 10 to be considered strong instrumental variables (IVs). Palindromes and incompatible alleles were eliminated from this analysis, as were SNPs with r^2^ > 0.001 and kb < 10,000, to prevent linkage disequilibrium effects. To further exclude potential confounders, SNPs linked to the outcome were examined using the LDlink database.^18^ Following these procedures, data analysis was conducted using the remaining SNPs (Supplementary Table S2).

### Statistical Analysis

NHANES analysis was meticulously weighted based on the officially supplied weighting variables to guarantee the national representativeness of the examined outcomes. For continuous variables, we applied the weighted mean ± standard deviation (SD) or weighted median (IQR), depending on their distributional characteristics. For binary variables, we utilised weighted percentages. We applied the weighted t-test or the weighted Wilcoxon rank-sum test for continuous variables and the weighted Chi-square test for categorical variables to compare the groups.

To explore the association between AGR and periodontitis, three weighted multivariate logistic regression models were constructed. Model 1 was unadjusted. After adjusting for age, gender, race, education, marital status, and PIR in Model 2, Model 3 took it a step further by adjusting for BMI, smoking, drinking, physical activity, diabetes, hypertension, CVD, CKD, and RA. AGR was changed from continuous to categorical variables for sensitivity analysis, and trend tests were run to evaluate how reliable the findings were. To examine the nonlinear association between AGR and periodontitis, the restricted cubic spline (RCS) function was employed. When a non-linear association was suggested by the RCS analysis, threshold effect analysis was further conducted using a two-piecewise linear regression model, and a likelihood ratio test was used to compare the standard linear regression model with the two-piecewise linear regression model. Logistic regression models and likelihood ratio tests were used for subgroup analyses and interaction testing, respectively.

To better reflect the diversity and uncertainty of the missing data in the covariates, five filler data sets were generated by the Gibbs Sampling method after a ‘warm-up’ process of 1,000 iterations and updated 1,000 times.^23,27^ To verify the robustness of the results, sensitivity analyses were conducted using data with missing covariate values removed. Although no *a priori* power calculation was performed because the NHANES database has a fixed sampling design and predetermined sample size, the large nationally representative sample and the narrow confidence intervals observed across models suggest that the study had sufficient statistical power to detect the observed associations.

To examine whether genetically predicted AGR was associated with periodontitis, we employed the inverse variance weighting (IVW) approach as our main tactic, with the MR-Egger, weighted median, weighted mode, and simple mode methods as supplements. We applied Cochran’s Q for heterogeneity analysis. This study additionally examined the IVW results for horizontal pleiotropy, which was assessed via MR-Egger intercept analysis to enhance the reliability of the results. Furthermore, this study evaluated the robustness of the findings by excluding outliers using the MR-PRESSO approach. To determine whether exposure influences the outcome through genetic variation or whether the outcome influences the exposure, the Steiger directionality test uses the covariance of the exposure and the outcome to assess the direction of the exposure-outcome relationship. This study additionally tested the effects of individual SNPs on the MR result using leave-one-out analysis to guarantee the analysis’s robustness.

All statistical analyses were performed using R software (version 4.2.2). A two-sided *p* value < 0.05 was considered statistically significant.

## RESULTS

### Results of the NHANES Study

Table 1 illustrates the baseline features of the subjects stratified by periodontitis status. This study comprised 10,094 participants, consisting of 5186 periodontitis patients and 4908 non-periodontitis individuals. Compared to the non-periodontitis group, the periodontitis group was predominantly composed of older males, Mexican Americans, non-Hispanic Blacks, individuals with lower education and income levels, and those who were widowed, divorced, or separated. This group also exhibited higher proportions of individuals with elevated BMI, smokers, and physically inactive subjects, as well as higher prevalence rates of hypertension, diabetes, CVD, CKD, and RA. Moreover, the periodontitis group was mainly distributed in the lower quartile ranges of AGR. Between-group differences were statistically significant (*p* < 0.05) for all the above variables except for alcohol use (*p* = 0.234).

**Table 1:** Weighted demographic features of participants, categorised by periodontitis status

Characteristic	Overall	Non-Periodontitis	Periodontitis	*p* value
	N = 136,995,911 n = 10,094	N = 79,194,070 n = 4908	N = 57,801,840 n = 5186	
**Age (years)**	50.00 (40.00–60.00)	46.00 (38.00–57.00)	54.00 (44.00–64.00)	< 0.001
**Gender (%)**				< 0.001
Male	49.34%	42.60%	58.57%	
Female	50.66%	57.40%	41.43%	
**Race (%)**				< 0.001
Mexican American	8.07%	5.60%	11.45%	
Non-Hispanic white	69.19%	75.44%	60.63%	
Non-Hispanic Black	10.19%	7.52%	13.83%	
Other race	12.55%	11.43%	14.09%	
**Education level (%)**				< 0.001
< High school	15.23%	9.22%	23.47%	
High school	21.02%	17.24%	26.19%	
> High school	63.75%	73.54%	50.34%	
**Marital status (%)**				< 0.001
Never married	10.14%	9.63%	10.84%	
Widowed/divorced/separated	20.19%	16.79%	24.84%	
Married/living with partner	69.67%	73.58%	64.32%	
**PIR (%)**				< 0.001
< 1.3	19.26%	13.21%	27.55%	
1.3–3.5	34.55%	30.73%	39.78%	
> 3.5	46.19%	56.06%	32.67%	
**BMI (%)**				< 0.001
< 25	26.94%	28.47%	24.84%	
25–29.99	35.61%	35.80%	35.35%	
≥ 30	37.45%	35.73%	39.81%	
**Physical activity (%)**				< 0.001
No	62.11%	59.05%	66.30%	
Yes	37.89%	40.95%	33.70%	
**Smoking status (%)**				< 0.001
Never	56.16%	63.79%	45.70%	
Former	26.39%	24.88%	28.47%	
Current	17.44%	11.33%	25.82%	
**Alcohol use (%)**				0.234
No	21.02%	20.23%	22.10%	
Yes	78.98%	79.77%	77.90%	
**Hypertension (%)**				< 0.001
No	59.88%	65.52%	52.14%	
Yes	40.12%	34.48%	47.86%	
**Diabetes (%)**				< 0.001
No	77.13%	81.87%	70.63%	
Prediabetes	8.39%	7.71%	9.33%	
Diabetes	14.47%	10.42%	20.03%	
**CVD (%)**				< 0.001
No	92.62%	95.22%	89.07%	
Yes	7.38%	4.78%	10.93%	
**CKD (%)**				< 0.001
No	86.61%	89.55%	82.59%	
Yes	13.39%	10.45%	17.41%	
**RA (%)**				0.040
No	95.82%	96.29%	95.17%	
Yes	4.18%	3.71%	4.83%	
AGR (g/L)	1.57 ± 0.29	1.61 ± 0.28	1.53 ± 0.29	< 0.001
**AGR category (%)**				< 0.001
Q1 (0.39–1.32)	17.91%	14.43%	22.68%	
Q2 (1.32–1.50)	21.74%	20.78%	23.07%	
Q3 (1.50–1.69)	26.98%	27.09%	26.84%	
Q4 (1.69–2.78)	33.36%	37.70%	27.41%	

AGR = albumin-to-globulin ratio, BMI = body mass index, CKD = chronic kidney disease, CVD = cardiovascular disease, PIR = poverty-to-income ratio, RA = rheumatoid arthritis.

N represents the weighted sample size, while n represents the unweighted sample size.

Normally and non-normally distributed continuous variables are expressed as Mean ± SD and Median (IQR), respectively. Categorical variables are presented as percentages.

*p* values compare the Non-Periodontitis and Periodontitis groups only. Continuous variables were compared using weighted t tests or weighted Wilcoxon rank-sum tests, and categorical variables using Rao–Scott corrected weighted Chi-square tests.

Table 2 demonstrates the results of the correlation study between AGR and periodontitis. It was found that AGR levels exhibited a significant negative correlation with the risk of developing periodontitis. This association was validated in all three models. The thoroughly adjusted model (model 3) showed a 48% decreased risk of developing periodontitis for each unit rise in AGR (OR = 0.52, 95% CI: 0.40–0.67). Furthermore, compared with individuals in the lowest quartile (Q1), those in the highest quartile (Q4) exhibited a 44% reduction in the risk of periodontitis (OR = 0.56, 95% CI: 0.46–0.68, *p* < 0.001). A significant decreasing trend in the risk of periodontitis was also observed (*p* < 0.001 for test of trend).

**Table 2:** Association between AGR and periodontitis, weighted

Exposure	Model 1		Model 2		Model 3	
	OR (95% CI)	*p* value	OR (95% CI)	*p* value	OR (95% CI)	*p* value
AGR	0.40 (0.33, 0.48)	< 0.001	0.53 (0.42, 0.67)	< 0.001	0.52 (0.40, 0.67)	< 0.001
**AGR quartile**						
Q1 (0.39–1.32)	Reference		Reference		Reference	
Q2 (1.32–1.50)	0.71 (0.60, 0.83)	< 0.001	0.76 (0.63, 0.91)	0.004	0.75 (0.62, 0.91)	0.005
Q3 (1.50–1.69)	0.63 (0.55, 0.72)	< 0.001	0.73 (0.61, 0.87)	< 0.001	0.70 (0.59, 0.84)	< 0.001
Q4 (1.69–2.78)	0.46 (0.40, 0.53)	< 0.001	0.57 (0.48, 0.68)	< 0.001	0.56 (0.46,0.68)	< 0.001
P for trend		< 0.001		< 0.001		< 0.001

AGR = albumin-to-globulin ratio, CI = confidence interval, OR = odds ratio.

Model 1: no variables were controlled for.

Model 2: controlled for gender, age, race, education level, marital status, and PIR.

Model 3: controlled for gender, age, race, education level, marital status, PIR, BMI, physical activity, smoking status, alcohol use, diabetes, hypertension, CKD, CVD, and RA.

We performed sensitivity analyses using data after removing missing values for covariates, and the results obtained were consistent with those described above (Table 3). Then, RCS analysis showed an L-shaped nonlinear relationship between AGR and the risk of periodontitis (*p* for non-linearity = 0.006) (Fig 3). This finding prompted us to conduct a threshold effect analysis (Table 4), which identified an inflection point at AGR = 1.79 (*p* for likelihood ratio test = 0.022). When AGR < 1.79, higher AGR was significantly associated with lower odds of periodontitis (OR = 0.37, 95% CI: 0.28–0.50), suggesting a 63% reduction in the odds of periodontitis for each one-unit increase in AGR. When AGR ≥ 1.79, the association tended to plateau (OR = 1.15, 95% CI: 0.54–2.45).

**Table 3:** Weighted association between AGR and periodontitis (complete-case analysis)

Exposure	Model 1			Model 2			Model 3	
	OR (95% CI)	*p* value		OR (95% CI)	*p* value		OR (95% CI)	*p* value
AGR	0.42 (0.33, 0.52)	< 0.001		0.55 (0.42, 0.71)	< 0.001		0.54 (0.41, 0.71)	< 0.001
**AGR quartile**								
Q1 (0.39, 1.32)	Reference			Reference			Reference	
Q2 (1.32, 1.50)	0.71 (0.59, 0.85)	< 0.001		0.74 (0.60, 0.93)	0.010		0.75 (0.60, 0.94)	0.016
Q3 (1.50, 1.70)	0.65 (0.56, 0.77)	< 0.001		0.72 (0.59, 0.89)	0.003		0.71 (0.58, 0.87)	0.002
Q4 (1.70, 2.82)	0.48 (0.40, 0.56)	< 0.001		0.58 (0.47, 0.71)	< 0.001		0.57 (0.46, 0.70)	< 0.001
*p* for trend		< 0.001			< 0.001			< 0.001

AGR = albumin-to-globulin ratio, CI = confidence interval, OR = odds ratio.

Model 1: no covariates were adjusted.

Model 2: adjusted for gender, age, race, education level, marital status, and PIR.

Model 3: adjusted for gender, age, race, education level, marital status, PIR, BMI, physical activity, smoking status, alcohol use, diabetes, hypertension, CKD, CVD, and RA.

**Table 4:** Threshold effect analysis of the association between AGR and periodontitis

Periodontitis	OR (95% CI)	*p* value
Model 1 Fitting model by standard linear regression	0.48 (0.39, 0.59)	< 0.001
Model 2 Fitting model by two-piecewise linear regression		
Inflection point	1.79	
AGR < 1.79	0.37 (0.28, 0.50)	< 0.001
AGR ≥ 1.79	1.15 (0.54, 2.45)	0.728
*p* for likelihood ratio test		0.022

AGR = albumin-to-globulin ratio, CI = confidence interval, OR = odds ratio.

The model was adjusted for gender, age, race, education level, marital status, PIR, BMI, physical activity, smoking status, alcohol use, diabetes, hypertension, CKD, CVD, and RA.

Figure 4 demonstrates the subgroup analysis of AGR-periodontitis associations. The analysis showed that age had a substantially modifying effect on the association between AGR and periodontitis (*p* for interaction = 0.008). Other subgroup analyses showed that gender, race, BMI, smoking status, hypertension, diabetes, CVD, CKD, and RA did not statistically modify the association between AGR and periodontitis (*p* for interaction > 0.05).

### Results of the MR Study

In investigating the causal link between AGR and periodontitis, we evaluated IVs based on established criteria and ultimately selected three SNPs for further research. IVW analysis revealed no significant causal relationship between AGR and periodontitis (OR = 0.76, 95% CI: 0.45–1.28, *p* = 0.304). Likewise, MR-Egger, weighted median, weighted mode, and simple mode did not demonstrate significant causal effects (Fig 5). Scatter plots illustrated the impact of AGR on periodontitis as assessed using several MR methodologies (Supplementary Fig S1a). Forest plots illustrated the estimated impact of each SNP on periodontitis (Supplementary Fig S1b).

Multiple sensitivity analyses were conducted to assess the reliability and robustness of the research. Cochran’s Q test demonstrated an absence of heterogeneity among the selected IVs (*p* > 0.05). The MR-Egger intercept and MR-PRESSO tests did not indicate horizontal pleiotropy (*p* > 0.05). Moreover, the funnel plot and leave-one-out method substantially mitigated the impact of outliers on the results (Supplementary Fig S1c and S1d). The Steiger test ultimately demonstrated a stronger association between IVs and AGR compared with the correlation between IVs and periodontitis.

## DISCUSSION

This study evaluated the relationship between AGR and periodontitis using NHANES data and two-sample MR analysis. In the cross-sectional analysis of 10,094 adults, higher AGR was associated with lower odds of periodontitis after adjustment for possible confounders. Sensitivity analyses provided more evidence of the results’ stability. RCS analysis suggested an L-shaped nonlinear pattern, and threshold effect analysis indicated that the inverse association was mainly observed when AGR was below 1.79, after which the association tended to plateau. In addition, subgroup analyses showed that the association was stronger among participants younger than 60 years. However, the MR analysis did not provide genetic evidence supporting this observational association.

Our findings are consistent with recent studies linking serum protein-related indicators to periodontitis. Xiang et al^38^ reported that lower serum albumin levels were associated with moderate-to-severe periodontitis, and Alshammari et al^3^ further showed that each 0.34 g/dL decrease in serum albumin corresponded to higher odds of periodontal disease. These findings suggest that serum protein alterations may reflect inflammatory and nutritional changes in periodontal disease. Compared with albumin alone, AGR may provide a broader protein-based indicator because it incorporates information from both albumin and globulin. More directly, recent NHANES-based studies by Chen et al^8^ and Huang et al^15^ reported an inverse association between AGR and periodontitis and suggested a nonlinear pattern in this relationship. In line with these studies, our analysis showed that higher AGR remained associated with lower odds of periodontitis after further adjustment for potential confounders.

The association between lower AGR and higher odds of periodontitis may be biologically plausible because AGR reflects the combined changes in albumin and globulin under inflammatory and nutritional stress. Periodontitis is driven by dysbiotic biofilms and an excessive host immune response, in which cytokine-mediated inflammation, oxidative stress, and neutrophil activation contribute to periodontal tissue destruction.^19,25^ In this inflammatory context, serum albumin may decrease not only because of impaired nutritional status but also because inflammatory illness can reduce albumin concentration through hepatic reprioritisation of protein synthesis and increased transcapillary albumin escape.^6^ Reduced albumin may therefore indicate diminished nutritional reserve, weakened antioxidant capacity, and a reduced ability to support tissue repair. At the same time, higher globulin levels may reflect immune activation, including increased immunoglobulins, complement components, and acute-phase reactive proteins.^20^ Thus, a lower AGR may reflect both albumin depletion related to inflammation and nutritional stress and globulin elevation related to immune-inflammatory activation. This signal may partly explain why individuals with lower AGR showed higher odds of periodontitis in the present study.

The L-shaped nonlinear association observed in this study is consistent with recent NHANES-based studies reporting that the AGR-periodontitis association was more evident at lower AGR levels and attenuated after a threshold.^8,15^ This pattern may be explained by the fact that low AGR reflects both decreased albumin and increased globulin. During inflammatory illness, albumin may decline because of hepatic reprioritisation of protein synthesis and increased transcapillary albumin escape, whereas globulin-related proteins may increase with immune activation and acute-phase responses.^6^ In periodontitis, dysbiotic biofilms and dysregulated host immune responses can promote cytokine production, oxidative stress, neutrophil activation, and periodontal tissue destruction.^21,41^ Therefore, when AGR is low, small changes in AGR may more sensitively reflect systemic inflammatory and nutritional imbalance related to periodontal disease. Once AGR reaches a relatively higher range, these imbalances may be less apparent, and further increases in AGR may provide limited additional information, which may explain why the inverse association tended to plateau after AGR reached approximately 1.79. Nevertheless, this threshold should be interpreted as a data-derived point rather than a clinical diagnostic cutoff, and longitudinal studies are needed to confirm this pattern.

Subgroup analysis suggested that age may modify the association between AGR and periodontitis. The inverse association was more pronounced among participants younger than 60 years, possibly because AGR in this group more directly reflects systemic inflammatory and nutritional imbalance related to periodontal inflammation. In older adults, age-related immune remodelling, including immunosenescence, inflammaging, and neutrophil dysfunction, may alter host responses to periodontal pathogens and complicate this association.^36,43^ Serum albumin in older individuals may also be influenced by chronic low-grade inflammation, comorbidities, nutritional status, and hepatic protein metabolism rather than periodontal inflammation alone.^2,6^ These factors may reduce the specificity of AGR as a marker of periodontal inflammatory burden in older adults.

This study has the following strengths: first, we pioneered the integration of cross-sectional and MR studies to investigate the relationship between AGR and periodontitis, thereby complementing the constraints of cross-sectional studies. Second, the NHANES study utilised the officially provided complex sampling weights for our analyses, allowing them to be effectively generalised to the entire noninstitutionalised US population. Nevertheless, this research suffers from the following constraints. First, in this cross-sectional study, despite covariate adjustment, residual confounding may persist due to uncontrolled variables. Second, some of the health information relied on self-reporting, which is susceptible to recall or reporting bias. Third, the populations encompassed in NHANES and MR are inconsistent, potentially introducing bias in the results and necessitating additional research for validation. Fourth, this study used the 2012 CDC/AAP case definition rather than the staging and grading framework of the 2018 EFP/AAP classification, which may limit further interpretation of periodontitis severity, complexity, and progression risk. Fifth, the number of SNPs associated with AGR is limited. In the future, it will be necessary to identify more relevant SNPs through larger-scale GWAS to enhance the robustness and statistical power of the findings.

## CONCLUSIONS

Our study indicated that AGR was inversely associated with periodontitis in an L-shaped pattern, with a threshold of approximately 1.79. This association was more pronounced among participants younger than 60 years. However, MR analysis did not provide genetic evidence supporting this observational association. Further longitudinal and interventional studies are needed to clarify the underlying mechanisms and validate these findings using more rigorous study designs.

### Acknowledgements

We deeply appreciate the personnel of the NHGRI GWAS Catalog, IEU OpenGWAS, and NHANES databases for their important contributions.

## REFERENCES

**Fig 1:** Process flow diagram for participant selection.AGR = albumin-to-globulin ratio, IQR = interquartile range, NHANES = National Health and Nutrition Examination Survey, Q1 = first quartile, Q3 = third quartile.

**Fig 2:** Framework of the research design.LD = linkage disequilibrium, MR = Mendelian randomisation, SNP = single nucleotide polymorphism.

**Fig 5:** The impact of AGR on periodontitis in MR analysis.AGR = albumin-to-globulin ratio, CI = confidence interval, OR = odds ratio.

**Fig 3:** Restricted cubic spline analysis of the relationship between AGR and periodontitis. Controlled for age, sex, race, education level, marital status, PIR, BMI, smoking, drinking, physical activity, and hypertension, diabetes, CKD, CVD, and RA.AGR = albumin-to-globulin ratio, CI = confidence interval, OR = odds ratio.

**Fig 4:** Subgroup analysis of the relationship between AGR and periodontitis. Adjustments were made for variables other than stratification variables, including gender, age, race, education level, marital status, PIR, BMI, physical activity, smoking status, alcohol use, diabetes, hypertension, CKD, CVD, and RA.










